# Molecular alterations in the TCR signaling pathway in patients with aplastic anemia

**DOI:** 10.1186/s13045-016-0261-6

**Published:** 2016-03-31

**Authors:** Bo Li, Lixing Guo, Yuping Zhang, Yankai Xiao, Mingjuan Wu, Lingling Zhou, Shaohua Chen, Lijian Yang, Xiang Lu, Yangqiu Li

**Affiliations:** Institute of Hematology, Medical College, Jinan University, Guangzhou, China; Department of Hematology, Guangzhou First Municipal People’s Hospital Affiliated to Guangzhou Medical College, Guangzhou, China; Key Laboratory for Regenerative Medicine of Ministry of Education, Jinan University, Guangzhou, China; Department of Hematology, Affiliated Hospital of Youjiang Medical University for Nationalities, Baise, China; Department of Hematology, First Affiliated Hospital, Jinan University, Guangzhou, China

**Keywords:** CD3ζ, CD28, CTLA-4, Cbl-b, CD3ζ 3′-UTR splice variant, SNP, Gene expression level, AA

## Abstract

**Background:**

A previous study has demonstrated a significantly increased CD3ζ gene expression level in aplastic anemia (AA). However, the mechanism underlying the upregulated CD3ζ mRNA expression level and that of T cell activation signaling molecules in AA patients remains unclear. Thus, we investigated the expression levels of the CD3ζ, CD28, CTLA-4, and Cbl-b genes, the SNP rs231775 in the CTLA-4 gene, and the distribution of the CD3ζ 3′-UTR splice variant in AA patients.

**Methods:**

CD3ζ 3′-UTR splice variants were identified in peripheral blood mononuclear cells (PBMCs) from 48 healthy individuals and 67 patients with AA [37 cases of severe aplastic anemia (SAA) and 30 cases of non-sever aplastic anemia (NSAA)] by RT-PCR. CD3ζ, CD28, CTLA-4, and Cbl-b gene expression was analyzed by real-time quantitative PCR. The SNP rs231775 in CTLA-4 gene was analyzed by PCR-RFLP.

**Results:**

CD3ζ and CD28 expression was significantly higher, while CTLA-4 and Cbl-b expression was significantly lower in AA patients compared with healthy individuals. Significantly higher CD3ζ expression was found in the NSAA subgroup compared with the SAA subgroup. 64 % of the AA samples had the same genotype (WT^+^AS^+^CD3ζ 3′-UTR); 22 % of the AA patients had a WT^+^AS^−^CD3ζ 3′-UTR genotype, and 14 % of the AA patients had a WT^−^AS^+^CD3ζ 3′-UTR genotype. The CD3ζ expression level of WT^−^AS^+^ subgroup was the highest in the SAA patients. A significantly higher frequency of the GG genotype (mutant type, homozygous) of SNP rs231775 in CTLA-4 gene was found in the AA patients. Positive correlation between the CTLA-4 and Cbl-b gene expression levels was found in healthy individuals with the AA and AG genotypes, but not in the AA patients.

**Conclusions:**

This is the first study analyzing the expression characteristics of the CD28, CTLA-4, and Cbl-b genes in AA. Our results suggest that aberrant T cell activation may be related to the first and second signals of T cell activation in AA. The GG genotype of SNP rs231775 in CTLA-4 gene might be associated with AA risk in the Chinese population. The characteristics of CD3ζ 3′-UTR alternative splicing may be an index for evaluating the T cell activation status in AA patients, particularly in SAA patients.

## Background

Aplastic anemia (AA) is a serious hematological disorder characterized by pancytopenia [1–3]. AA is an immune-mediated destruction of hematopoietic cells caused by abnormally activated T cells for most cases [4, 5]. Our previous study has shown that, in addition to abnormal distribution and clonal expansion of the T cell receptor (TCR) repertoire, there is significantly higher CD3ζ expression in T cells in AA patients [6]. An abnormal CD3ζ gene expression level may directly represent a characteristic of abnormal T cell activation.

T cell recognition of antigenic peptide/major histocompatibility complexes plays a pivotal role in the initiation and regulation of the adaptive immune response [7–9]. TCR activation plays a crucial role in T cell function. The TCR itself does not possess signaling domains. Instead, the TCR is non-covalently coupled to a conserved multisubunit signaling apparatus, i.e., the CD3 complex, which comprises the CD3εγ, CD3εδ, and CD3ζζ dimmers [10]. However, the TCR/CD3 signaling complex alone is insufficient for antigen-specific T cell responses and a second pathway, co-stimulatory signaling, is required for T cell immune responses. The co-stimulatory signaling molecule CD28 that is found on T cells must bind B7-1 and B7-2, which are expressed on antigen presenting cells (APCs), to trigger T cell activation [11, 12]. Upon T cell activation, cytotoxic T-lymphocyte antigen-4 (CTLA-4) is induced and outcompetes CD28 for B7-1 and B7-2 ligands, thereby preventing excessive T cell expansion [13, 14]. This mechanism provides a key checkpoint in the regulation of T cell immunity [15, 16]. The SNP rs231775, A > G transition mutation which is located at exon 1 in the CTLA-4 gene, has been reported to potentially influence the inhibitory function of CTLA-4 by reducing its cell surface expression [17, 18].

Optimal T cell activation requires signaling through the TCR and the CD28 co-stimulatory receptor. CD28 co-stimulation is believed to set the threshold for T cell activation. Casitas B-lineage lymphoma proto-oncogene-b (Cbl-b), a RING finger E3 ubiquitin-protein ligase, is involved in CD28-dependent T cell activation [19]. Results from T cell activation assays in vitro have shown that CD28 co-stimulation promotes TCR-induced Cbl-b degradation, whereas CTLA-4-B7 interaction potentiates TCR-induced Cbl-b re-expression [20].

Expression of the CD3ζ gene is regulated at the transcriptional, posttranscriptional, and posttranslational levels [21]. As previously described, there are two isoforms of the CD3ζ 3′-UTR: a wild type (WT) isoform (906 bp) and an alternatively spliced (AS) isoform (344 bp). Abnormal CD3ζ expression was found in T cell from SLE patients, and this may be associated with decreased stability and translation of CD3ζ mRNAs that contain AS CD3ζ 3′-UTRs [22]. However, the mechanism of upregulating the CD3ζ mRNA expression level in AA patients is unclear.

Thus, we investigated the expression level of the CD28, CTLA-4, Cbl-b, and CD3ζ genes, the SNP rs231775 in CTLA-4 gene, CD3ζ-regulating factors, and the distribution of the CD3ζ 3′-UTR splice variant. We concluded that analysis of these factors may facilitate the comprehensive understanding of the abnormal T cell immune characteristics of AA.

## Results

### The expression levels of CD3ζ, CD28, CTLA-4, and Cbl-b in AA

**Fig. 1:**
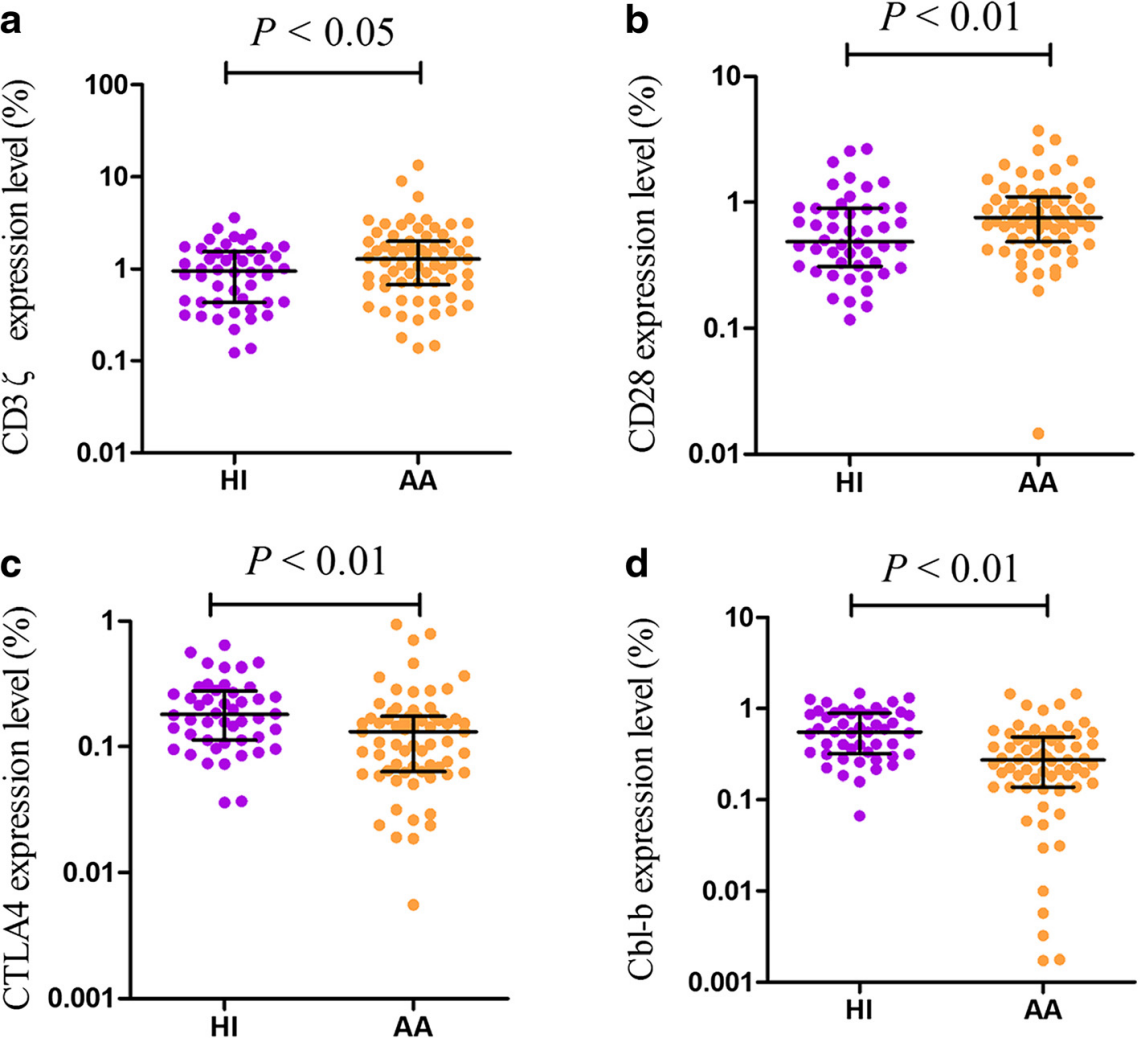
The relative gene expression levels of the CD3ζ, CD28, CTLA-4, and Cbl-b in AA and HIs. **a** The relative gene expression levels of CD3ζ in AA and HIs; **b** the relative gene expression levels of CD28 in AA and HIs; **c** the relative gene expression levels of CTLA-4 in AA and HIs; **d** the relative gene expression levels of Cl-b in AA and HIs

**Fig. 2:**
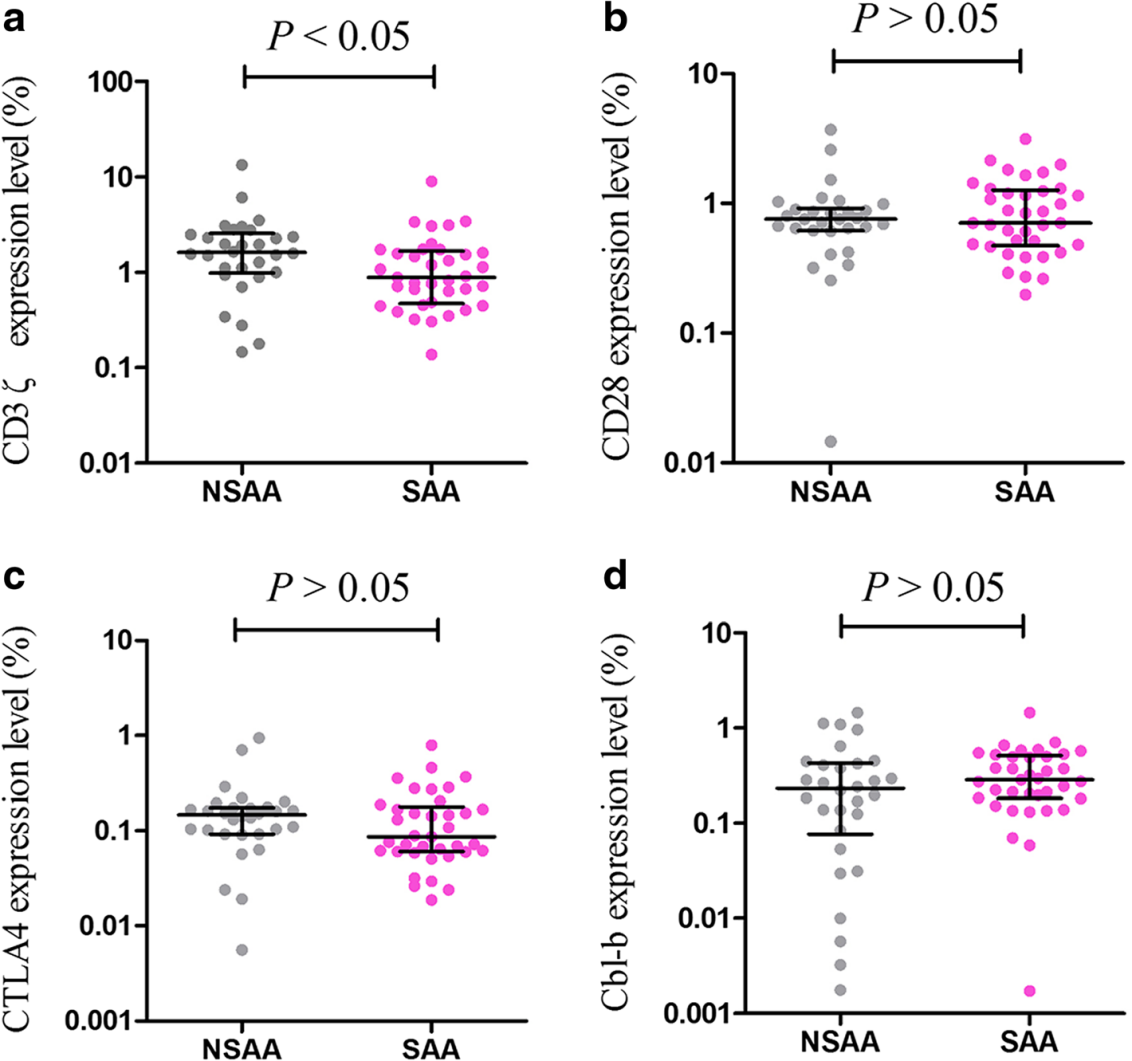
The relative gene expression levels of CD3ζ, CD28, CTLA-4, and Cbl-b in SAA and NSAA. **a** The relative gene expression levels of CD3ζ in SAA and NSAA; **b** the relative gene expression levels of CD28 in SAA and NSAA; **c** the relative gene expression levels of CTLA-4 in SAA and NSAA; **d** the relative gene expression levels of Cbl-b in SAA and NSAA

The expression levels of the CD3ζ, CD28, CTLA-4, and Cbl-b genes in cDNA from PBMCs from 67 AA patients before treatment and 48 healthy individuals were quantitatively assessed by real-time polymerase chain reaction (PCR) using the SYBR Green I technique. The results demonstrated an increased expression level for CD28 (median 0.75) and CD3ζ (median 1.27) in AA patients compared with healthy individuals (CD28 0.49, *P <* 0.01; CD3ζ 0.95, *P <* 0.05; Fig. 1), while significantly decreased CTLA-4 (median 0.13) and Cbl-b (median 0.27) expression was found (CTLA-4 0.18, *P <* 0.01; Cbl-b 0.55, *P <* 0.01; Fig. 1). Significantly increased CD3ζ expression (median: 0.13) was found in the non-severe aplastic anemia patients (NSAA) compared with sever aplastic anemia patients (SAA, median: 0.18, *P <* 0.05). There were no significant differences in CD28, CTLA-4, and Cbl-b expression between the SAA and NSAA groups (Fig. 2).

### The expression characteristics of the CD3ζ gene with different CD3ζ 3′-UTR isoforms in AA

**Fig. 3:**
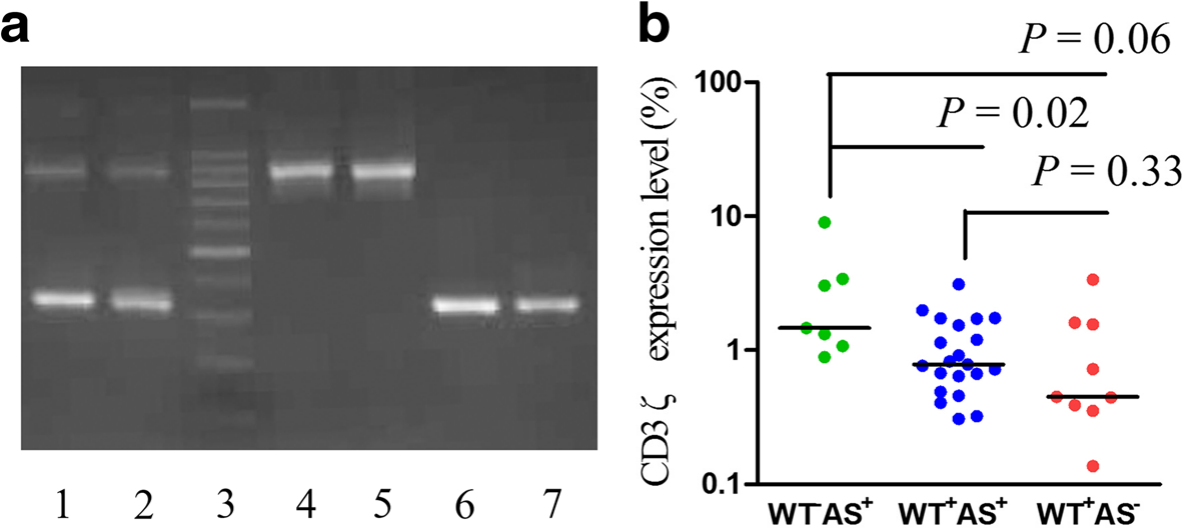
CD3ζ 3′-UTR splicing variant isoforms in AA and HIs. **a** The WT and AS isoforms could be detected in all of the healthy individuals (lane 1), 64 % of the AA patients contained WT^+^AS^+^CD3ζ 3′-UTR (lane 2), 22 % of the AA patients had only the WT^+^AS^−^CD3ζ 3′-UTR (lanes 4 and 5), and 14 % of the AA patients had only the WT^-^AS^+^CD3ζ 3′-UTR (lanes 6 and 7). Lane 3 is a 100 bp DNA ladder; **b** the expression level of the CD3ζ gene with different CD3ζ 3′-UTR splicing variant isoforms in SAA

Two types of CD3ζ 3′-UTR splicing variants, WT and AS CD3ζ 3′-UTR, were detected in all of the healthy individuals. However, a significantly lower frequency of the WT^+^AS^+^CD3ζ 3′-UTR genotype was detected in the AA patients (43 cases, *P <* 0.01), and a significantly higher frequency of the WT^+^AS^-^CD3ζ 3′-UTR (15 cases, *P* < 0.01) and WT^−^AS^+^CD3ζ 3′-UTR (9 cases, *P <* 0.01) genotypes were detected in the AA patients (Fig. 3a). There were no significant differences in the frequencies of the three types of CD3ζ 3′-UTR between the SAA and NSAA patients although the frequency of the WT^-^AS^+^CD3ζ 3′-UTR was high in the SAA patients (19 %) compared with that of the NSAA patients (7 %), while the WT^+^AS^+^CD3ζ 3′-UTR frequency was low in the SAA patients (57 %) compared with that of the NSAA patients (73 %).

Based on the CD3ζ 3′-UTR isoform expression, we divided the 67 AA cases into three subgroups: WT^+^AS^+^, WT^+^AS^−^ and WT^−^AS^+^. There were no significant differences in the CD3ζ expression among the three subgroups. To better comprehend the difference in the CD3ζ expression level between the SAA and NSAA patients, we compared the CD3ζ expression level in the WT^+^AS^+^, WT^+^AS^−^, and WT^−^AS^+^ subgroups in the SAA and NSAA patients. A significantly increased CD3ζ expression level (median 1.46) was found in the WT^−^AS^+^ subgroup compared with the WT^+^AS^+^ (median 0.78, *P* < 0.05) and WT^+^AS^−^ subgroups (median 0.45, *P =* 0.06) in the SAA patients. However, there were no significant differences in the CD3ζ expression level among the three subgroups in the NSAA patients (Fig. 3b).

### The genotype frequency for SNP rs231775 in CTLA-4 gene in AA

**Fig. 4:**
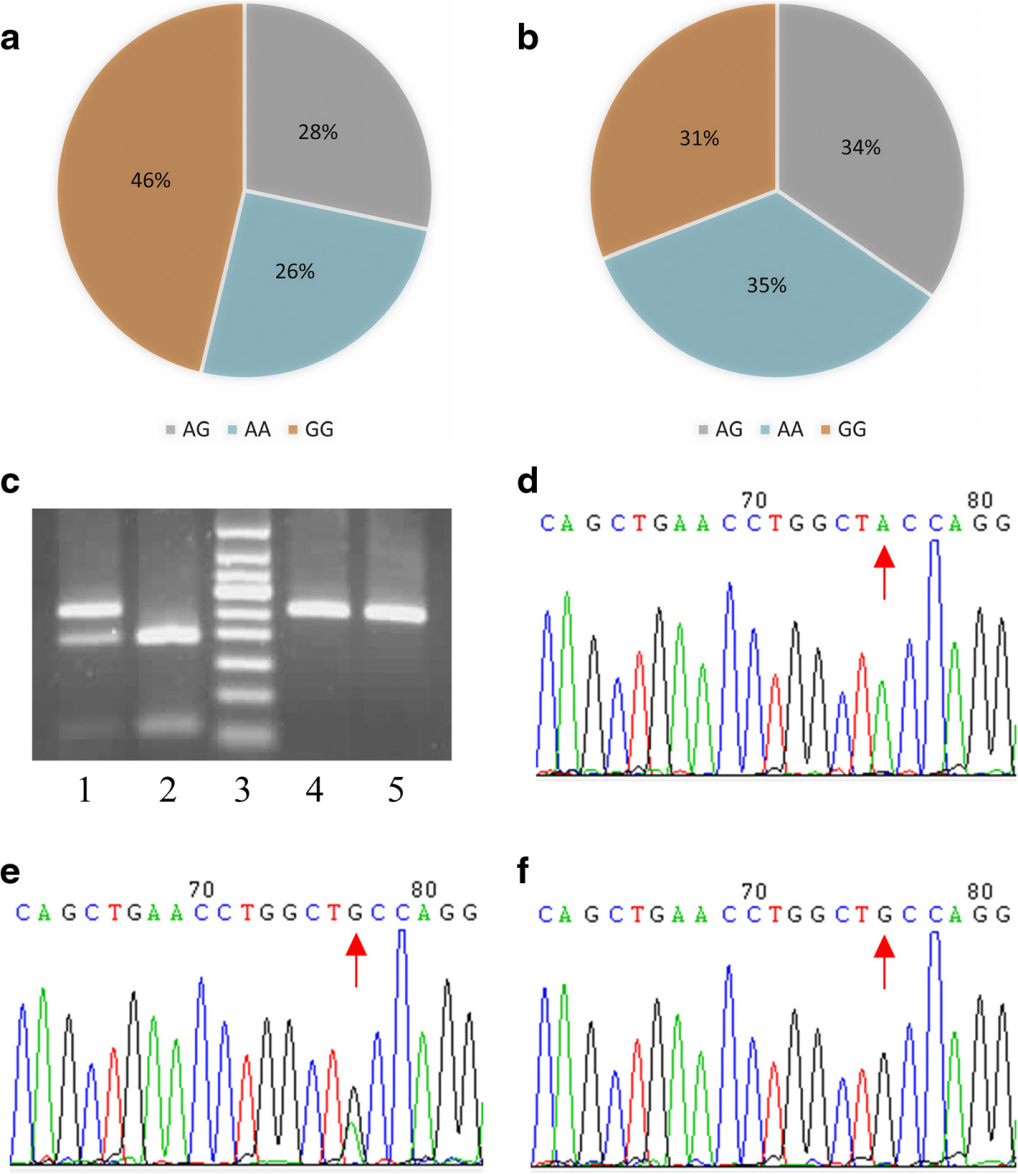
The genotype characteristic of SNP rs231775 in CTLA-4 gene in AA and HIs. **a** Distribution of the genotype of SNP rs231775 in AA patients (*n* = 67); **b** distribution of the genotype of SNP rs231775 in HIs (*n* = 84); **c** agarose gel electrophoresis results for SNP rs231775 in CTLA-4 gene, lane 1 is the AG genotype, lane 2 is the GG genotype, lane 3 is a 50 bp DNA ladder, lane 4 is the AA genotype, and lane 5 is a PCR product without TseI digestion; **d** sequencing results of AA genotype; **e** sequencing results of AG genotype; **f** sequencing results of GG genotype; the red arrow indicates the single-nucleotide polymorphism sites

The genotype distribution for SNP rs231775 in CTLA-4 gene in the AA patients (67 cases) and healthy individuals (84 cases) is shown in Fig. 4a, b. Gel electrophoresis results from TseI digests and the sequences of the different genotypes of SNP rs231775 in CTLA-4 gene are shown in Fig. 4c–f. AA patients had a borderline significantly higher frequency of the GG homozygous genotype compared with healthy individuals (46 % vs. 31 %, OR = 1.92, 95 % CI = 0.99–3.74, *P =* 0.05). AA patients had a significantly higher frequency of the G allele of SNP rs231775 in CTLA-4 gene compared with healthy individuals (60 % vs. 48 %, OR = 0.61; 95 % CI = 0.39–0.97, *P* = 0.03). Analysis of the AA patients with the different genotypes for correlations with clinical parameters including age of onset, gender, severity of AA, and laboratory parameters was performed. However, no clinical or laboratory parameters were found to have a significant association with different genotypes of SNP rs231775 in CTLA-4 gene. In addition, the CTLA-4 expression level in the AA patients and healthy individuals had no significant association with the genotypes of SNP rs231775 in CTLA-4 gene.

### Correlation of the relative expression of CTLA-4 and Cbl-b in AA

**Fig. 5:**
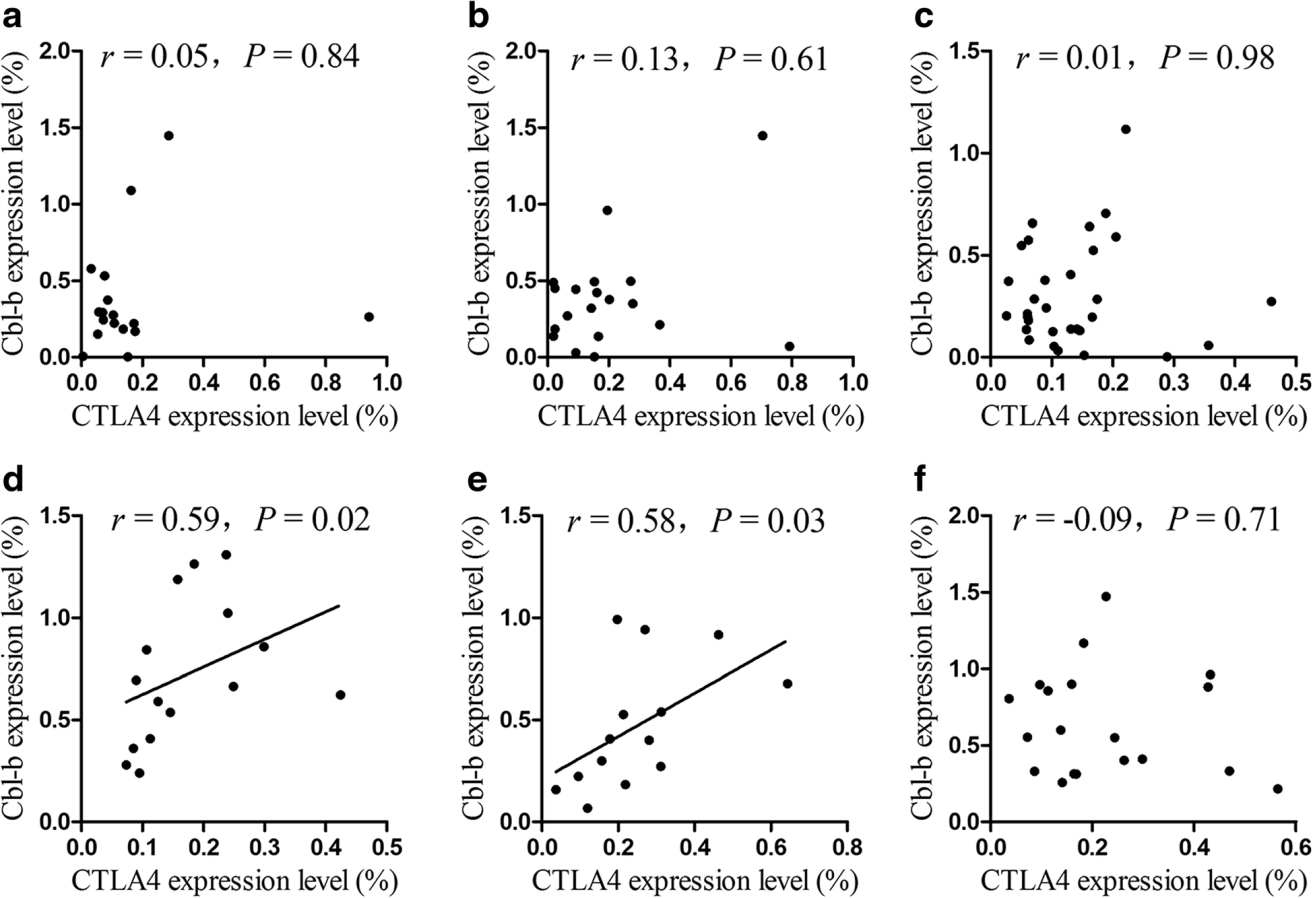
Correlation analysis of the CTLA-4 and Cbl-b expression levels for different genotypes of SNP rs231775 in CTLA-4 gene in AA and HIs. **a** CTLA-4 and Cbl-b in AA with the AA genotype, **b** CTLA-4 and Cbl-b in AA with the AG genotype, **c** CTLA-4 and Cbl-b in AA with the GG genotype, **d** CTLA-4 and Cbl-b in HIs with the AA genotype, **e** CTLA-4 and Cbl-b in HIs with the AG genotype, and **f** CTLA-4 and Cbl-b in HIs with the GG genotype

It has been reported that CTLA-4 regulates Cbl-b at the transcriptional level. We next estimated the correlation between the CTLA-4 and Cbl-b gene expression levels in AA patients and healthy individuals. There was a significant correlation between the expression levels of the CTLA-4 and Cbl-b genes in healthy individuals with the AA (rs = 0.59, *P* = 0.02) and AG (rs = 0.58, *P* = 0.03) genotypes, while there was no significant correlation between the expression levels of the CTLA-4 and Cbl-b genes (rs = −0.09, *P* = 0.71) in healthy individuals with the GG genotype (Fig. 5). In all of the patients with the various genotypes, there was no significant correlation between the expression levels of the CTLA-4 and Cbl-b genes.

## Discussion

AA is an immune-mediated disease in which T cell target hematopoietic cells [23, 24]. The precise mechanism of T cell activation in AA pathogenesis remains unknown. TCR signaling plays an important role in T cell activation [25, 26]. In this study, we analyzed the expression of TCR signaling molecules and related factors that are involved in mediating TCR signaling in AA patients.

T cell activation requires the delivery of at least two signals. The first signal is antigen-specific and MHC-TCR ligation, and the second is a co-stimulatory signal [13, 27, 28]. Co-stimulatory signals occur via the binding of CD28, which is located on T cell, with B7 family members on APCs. Initial activation of T cell after antigen exposure is mediated by the CD28/B7 interaction and leads to the proliferation and differentiation of effector T cell [29]. Co-stimulation cannot proceed unchecked; otherwise, an overwhelming immune response will ultimately ruin the host. The co-stimulatory system consists of peculiar mechanisms that can dampen T cell activation signals, resulting in immune homeostasis. CTLA-4, an inhibitory signaling molecule that prevents T cell activation, becomes expressed on activated T cell when T cell activation has reached its peak [30, 31]. CD28 and CTLA-4 control the threshold for T cell activation by regulating the level of Cbl-b expression [19, 20].

In this study, we analyzed the expression characteristics of the CD3ζ, CD28, CTLA-4, and Cbl-b genes, and our data indicate significantly decreased CTLA-4 and Cbl-b and increased CD3ζ and CD28 expression in AA patients. These results suggest that there may be aberrant T cell activation, which may be related to the first and second signals of the T cell activation signaling molecules in AA. However, do the other signaling molecules of TCR signaling such as CD3γ, CD3δ, CD3ε, and ZAP-70 [32, 33] also have similar change trend in AA, it is needed further investigation, and it is suggested to characterize the alteration of gene expression profile. Downregulated Cbl-b gene expression in AA suggests that the threshold for T cell activation is different from that in healthy individuals. There is variation in the CD3 gene expression level in SAA and NSAA patients, which indicates that an aberrant T cell activation is more obvious in NSAA patients and may help distinguish between SAA and NSAA patients for immunosuppressive treatments in the clinic.

It is known that CD3ζ is the key regulator of the TCR/CD3 complex in T cell activation [34, 35]. The increased CD3ζ expression in AA samples indicated abnormal T cell activation at least in some subsets of T cell in the patients, while a differently significant CD3ζ gene expression pattern was found for SAA and NSAA in this study. However, the underlying mechanism remains unclear because a previous study showed that different distributions of the CD3ζ 3′-UTR isoforms may affect CD3ζ and its related gene expression. Therefore, we compared the CD3ζ expression characteristics in subgroups containing different combinations of the CD3ζ 3′-UTR isoforms.

In general, the WT and AS 3′-UTR play different roles in CD3ζ transcript stability and generation of the CD3ζ protein [36]. In this study, we first characterized the distribution of the WT and AS 3′-UTRs in AA patients. Interestingly, we found that only 64 % of the AA samples expressed WT^+^AS^+^CD3ζ 3′-UTR, which was thought to be the normal genotype. We also found two different subgroups, WT^+^AS^−^CD3ζ 3′-UTR (22 %) and WT^−^AS^+^CD3ζ 3′-UTR (14 %), in the AA patients, which may be related to the change in the CD3ζ level in the patients. Because we could not find a difference in the CD3ζ expression level in the total AA samples, we further compared the SAA and NSAA groups. Notably, an increased CD3ζ expression level was found in the WT^−^AS^+^ CD3ζ 3′-UTR SAA group compared with both the WT^+^AS^+^ CD3ζ 3′-UTR and WT^+^AS^−^ CD3ζ 3′-UTR SAA groups. Interestingly, there was no significant difference in the CD3ζ expression level between the WT^+^AS^+^, WT^−^AS^+^, and WT^+^AS^−^ subgroups for the NSAA patients. Therefore, there may be a difference in the regulation of the CD3ζ expression level and T cell activation between SAA and NSAA. At a minimum, it is thought that the CD3ζ 3′-UTR isoforms may influence the CD3ζ expression level in T cells from SAA, which may be one reason for the abnormal T cell activation.

It has been reported that aberrant CD3ζ expression may be associated with the decreased stability and translation of CD3ζ mRNAs with an AS CD3ζ 3′-UTR in SLE [22, 36]. In this study, the CD3ζ gene expression level in some SAA patients was thought to be affected by different splicing variants of the CD3ζ 3′-UTR. As our results demonstrated, there was an increased CD3ζ expression level in SAA patients with the WT^-^AS^+^ CD3ζ 3′-UTR genotype, and whether this is feedback regulation remains an open question. The T cell activation function in SAA patients will be examined in the future to evaluate the influence of the CD3ζ 3′-UTR isoforms. To characterize the positive and negative regulatory factors of the TCR signal pathway, it is necessary to analyze the characteristics of the negative factors e.g., CTLA-4 and Cbl-b. It has been reported that SNP rs231775 in CTLA-4 gene is associated with an increased frequency of autoimmune diseases such as Graves’ disease, autoimmune hypothyroidism, type I diabetes, and multiple sclerosis [37–39]. ]s To determine whether this SNP also plays a role in AA, we analyzed its distribution in AA samples. To our knowledge, this is the first study to analyze this SNP in Chinese AA patients. Interestingly, we found that the homozygous GG mutant was significantly higher in AA patients, indicating that this CTLA-4 variant may have an association with susceptibility to developing AA. However, this result is in contrast with a report by Svahn J et al. who demonstrated that there were no significant differences in the genotype or allele frequency of SNP rs231775 in CTLA-4 gene between AA patients and healthy individuals in the Caucasian population [40]. However, the CTLA-4 mRNA expression level had no significant association with the genotype of SNP rs231775 in CTLA-4 gene in this study because SNP rs231775 is associated with the protein level [17, 18]. Further study is needed to analyze the CTLA-4 protein level in T cell from AA patients to confirm this result.

CTLA-4 regulates TCR signals via Cbl-b, and it plays an important role in regulating it at the transcriptional level [20]. However, little is known about the mRNA expression pattern of CTLA-4 and Cbl-b in AA patients. In this study, we found a significant positive correlation between CTLA-4 and Cbl-b in healthy individuals with the AA and AG genotypes of SNP rs231775 in CTLA-4 gene but not in those with the GG genotype of SNP rs231775 in CTLA-4 gene. However, there was no significant correlation between CTLA-4 and Cbl-b in any of the patients. These results indicate that CTLA-4 loses its proper regulatory role of Cbl-b expression at the transcriptional level in AA patients.

## Conclusion

In conclusion, this is the first study to analyze the expression characteristics of the CD28, CTLA-4, and Cbl-b genes, the distribution of the CD3ζ 3′-UTR splice variant in AA patients, and the SNP rs231775 in CTLA-4 gene in Chinese AA patients. We found that aberrant T cell activation may be associated with either the first or second T cell activation signaling molecules in AA. Different CD3ζ expression patterns in SAA and NSAA patients suggest different abnormal alternative CD3ζ pathway patterns in SAA and NSAA patients. The mutant type homozygous GG and G allele for SNP rs231775 in CTLA-4 gene might be associated with AA risk for the Chinese population.

## Methods

### Samples

The AA group consisted of 67 patients with newly diagnosed AA (36 cases with SAA and 31 with NSAA, including 37 males and 30 females; median age 34 years, range 9–78 years). The information and clinical data of the patients are described in Table 1. Forty-eight healthy individuals (21 males and 27 females; median age 35.5 years, range 20–80 years) were used for detecting CD3ζ, CD28, CTLA-4, and Cbl-b genes expression levels and CD3ζ 3′-UTR isoform, and 84 healthy individuals (40 males and 44 females; median age 33 years, range 6–88 years) were used for detecting for SNP rs231775 in CTLA-4 gene. AA diagnoses were established by bone marrow biopsy and peripheral blood counts. All of the procedures were performed according to the guidelines of the Medical Ethics committee of the health bureau of the Guangdong Province of China. Peripheral blood mononuclear cell (PBMC) isolation, RNA and DNA extraction, and cDNA synthesis were performed according to the manufacturer’s instructions.

**Table 1:** The demographic and clinical characteristics of AA patients

Patients characteristics (*n* = 67)	No. (%)
Age (years)	Median 34.0
	Range 9.0–78.0
Gender	
Male	37(55.2)
Female	30(44.8)
Severity of AA	
AA	31 (46.3)
SAA	36 (53.7)
Clinical data	
Hb (g/L)	Median 65.0
ANC (10^9^/L)	Median 0.38
PLT (10^9^/L)	Median 11.5

*Hb* hemoglobin, *ANC* absolute neutrophil count, *PLT* platelet

### RT-PCR for CD3ζ 3′-UTR isoform

The primer specificity for CD3ζ 3′-UTR amplification was as follows: forward 5′-CAGCCAGGGGATTTCACCACTCAAAG-3′ reverse 5′-CCCTAGTACATTGACGGGTTTTTCCTG-3′ [41]. The amplification was carried out after initial denaturation at 94 °C for 4 min, 30 cycles at 94 °C, 45 s; 67 °C, 1 min; 72 °C for 2 min; and a final extension at 72 °C for 7 min. After completion of the PCR, 20 μl of the PCR products were electrophoresed on a 1.5 % agarose gel.

### Real-time relative quantitative PCR for the CD3ζ, CD28, CTLA-4, and Cbl-b genes

Real-time PCR using the SYBR Green I method [42, 43] was used to examine the CD3ζ, CD28, CTLA-4, and Cbl-b genes with cDNA obtained from the PBMCs of AA patients and healthy individuals. The CD3ζ and β_2_M primer sequences and PCR conditions were previously described [26]. The other primer sequences were as follows: CD28: 5′-GAAACACCTTTGTCCAA GTC-3′ and 5′-GGGGAGTCATGTTCATGTAG-3′, CTLA-4: 5′-GTCAGCCTGCCGAAGC ACT-3′ and 5′-GTCAGCCTGCCGAAGCACT-3′, and Cbl-b: 5′-CCCTGGAATTGACCATTGGG-3′ and 5′-ACTTGCCCAACTCAGTGAGAA-3′. The real-time PCR reactions were performed in a total volume of 25 μl containing approximately 1 μl cDNA, 0.5 μM of each primer pair, and 11.25 μl 2.5× RealMasterMix (Tiangen, Beijing). After an initial denaturation at 95 °C for 15 min, 40 cycles of the following procedure were performed using an MJ Research DNA Engine Opticon 2 PCR cycler (BIO-RAD, Hercules, CA, USA): 15 s at 95 °C and 30 s at 60 °C followed by 1 s at 80 °C for plate reading. The 2(^–∆CT^) method was used to analyze the genes of interest relative to an internal control gene.

### Polymerase chain reaction and restriction fragment length polymorphism (RFLP)

The SNP rs231775 in CTLA-4 gene exon 1 were amplified using the following primers: 5′-CACATGTGTAATACATATCTGGG-3′ and 5′-TTGCAGAAGACAGGGATG AAGA-3′. The polymerase chain reaction (PCR) mixture contained 100 ng of genomic DNA, 0.1 mM each of deoxynucleotide triphosphates, 12.5 pmol each of the primers, and 1 U Taq polymerase (Applied Biosystems, Foster City, CA, USA) in a 25 μl final volume. The samples were heated at 95 °C for 2 min followed by 35 cycles of 94 °C for 60 s, 60 °C for 60 s, and 72 °C for 60 s with a final extension at 72 °C for 5 min. The PCR products (50 μl) were digested overnight with the restriction enzyme TseI (NEB, UK) according to the manufacturer’s protocol, and they were analyzed by 2.5 % agarose gel electrophoresis. The digestion of PCR products in the presence of the restriction sites is indicative of the presence of the G allele. The finding of a single band of 250 bp is indicative of the AA genotype, whereas the GG genotype is indicated by the finding of two bands at 189 and 61 bp. On the other hand, the AG genotype is characterized by the appearance of three bands: one at 250 bp and the others at 189 and 61 bp. The different genotypes were successfully confirmed by DNA sequencing.

### Statistical analysis

The Mann-Whitney test was performed to compare each gene expression level between different groups. Spearman correlation analyses were used to estimate correlations between different gene expression levels. Fisher’s exact test was used to compare genotypes or allelic frequencies between different groups. A *P* < 0.05 was considered to be statistically significant.
